# The application of magnetic anchoring traction device in assisting donor liver bench surgery in classic orthotopic liver transplantation

**DOI:** 10.1186/s12876-022-02473-w

**Published:** 2022-11-16

**Authors:** Jiashu Song, Yue Wang, Xianming Cai, Jianhua Shi, Liangshuo Hu, Pengkang Chang, Wei Zhang, Bo Tang, Yi Lv, Xiaogang Zhang

**Affiliations:** 1 National Local Joint Engineering Research Center for Precision Surgery and Regenerative Medicine, First Affiliated Hospital of Xi’an Jiaotong University, No. 76, West Yanta Road, Xi’an, 710061 Shaanxi Province China; 2 Department of Hepatobiliary Surgery, First Affiliated Hospital of Xi’an Jiaotong University, No. 277, West Yanta Road, Xi’an, 710061 Shaanxi Province China; 3 Xi’an Magnetic Medical Technology Co., Ltd., Xi’an, 710061 China

**Keywords:** Magnetic anchor traction, Bench surgery, Liver transplantation

## Abstract

**Objective:**

To explore the clinical application of a newly developed magnetic anchoring traction (MAT) system in the liver bench trimming and transplantation surgery.

**Background:**

The conventionally limited space, vision, and exposure have always been a challenge for the quality of surgery in the liver bench trimming due to the fact that the exposure depends largely on the experience of surgeon. To deal with this problem, a MAT system is developed as an alternative support to enhance exposure. The preliminarily experiments on animals verified its feasibility and reliability in the practical use, and its clinical application and effects were examined in the present research.

**Methods:**

A total of 20 DCD (donation of cardiac death) donor livers were collected and divided evenly between the magnetic anchor traction (MAT) assisted group (n = 10) and the manual assisted group (n = 10). The results and quality assessment from experts about the liver bench surgery performed by two groups were examined and compared.

**Results:**

The MAT system can be employed effectively to compete and replace the manual assistance to achieve a better exposure in the liver bench trimming. No statistical difference was found regarding the baseline data between the MAT and the manual groups. In the inferior vena cava and hepatic artery trimming, the MAT group outperformed the manual group remarkably in many aspects. The surgery time for liver bench shortened considerably after a quick grasp of MAT skills by surgeons.

**Conclusion:**

The MAT system provides a more stable, reliable and qualified local exposure in the liver bench surgery, and can preferably be employed to replace the manual assistance in the procedures of liver transplantation.

## Background

Liver transplantation is broadly accepted as an effective option in dealing with a range of irreversible acute and chronic liver diseases [1–3]. After decades of clinical application, liver transplantation has increasingly developed into an even more matured, stabilized and standard approach in the treatment of terminal patients with liver diseases [4]. In practice, the donor liver needs to be obtained timely, trimmed and assessed simultaneously before being transplanted for the recipient [3]. And the donor extraction and trimming techniques, as well as time limit for surgery tend to be the prerequisites and guarantee for the desirable outcome of transplantation and follow up recovery [5]. However, the shortage of specialists, the extremely high training costs, along with the needs for more manual assistance and operating sites, the inconvenience caused by limited space, vision, and exposure, often present a great challenge for the quality of operation [6–11]. For all these problems, the emerging of the magnetic anchoring traction (MAT) assisted system or simply MAT, formulated and employed in the present clinical research, can be a promising solution.

This paper reports and specifies the application and strength of the MAT system in the clinical liver bench trimming surgery and transplantation. The system, which combines the magnetic base of the liver trimming table with the anchoring traction grasper, can provide a more stable, reliable and qualified exposure for the surgical performance, and can be used preferably to replace the manual assistance and enhance explore in the operation.

## Methods

The magnetic anchoring traction (MAT) has been registered as the first-class medical equipment (Mechanical equipment No. 20190006, Shaanxi Province) after the preliminary safety and feasibility experiment on animals. To further verify and address the safety and effectiveness of MAT in the clinical treatment, a series of research registered at Clinicaltrials.gov (02/12/2019, NCT04182256) have been conducted in the liver transplantation with an approval from the ethics committee of First Affiliated Hospital of Xi’an Jiaotong University. All cases for the experiment were required to sign the informed consent for clinical research before surgery. A total of 20 DCD (donation of cardiac death) donor livers were collected from October 1st to December 31st in 2020.

### Inclusion criteria

1. The DCD donor aged 18 or above
2. The DCD donor agreed to donate livers.

### Exclusion criteria

1. Bench surgery in split liver transplantation
2. Bench surgery in reduced-size liver transplantation
3. Supra-hepatic inferior vena cava is too short
4. Infra-hepatic inferior vena cava is too short

### The liver bench surgery

The donor livers were divided evenly between the MAT assisted group (n = 10) and the manual assisted group (n = 10). In the MAT group, the magnetic anchoring traction system was employed to assist the surgeon in the vascular exposure operation (the MAT device is used the same way as it is used in animal experiments); in the manual group, the vascular exposure was assisted manually. Twenty cases of bench surgery were performed by two surgeons.

The bench surgery processed as follows:

1. Infra-hepatic inferior vena cava trimming: Firmly ligated the right adrenal branch of the infra-hepatic inferior vena cava and each small branches.
2. Supra-hepatic inferior vena cava trimming: Firmly ligated the diaphragmatic vein, opened the diaphragm ring, and trimmed the diaphragm around the supra-hepatic inferior vena cava to ensure that the length of the supra-hepatic inferior vena cava was more than 2 cm.
3. Performed a venous leakage test: Sutured the break with 5-0 prolene to ensure that there was no leakage.
4. Portal vein trimming: The portal vein pipeline was drained of air bubbles, cut off the pipeline at the distal end of the pipeline switch, leaving enough length of the portal vein, and ligating all branches to the hepatic portal.
5. Hepatic artery trimming: Dissected the free superior mesenteric artery and checked whether there was an ectopic right hepatic artery at a distance of about 2 cm from the start. Dissected the celiac trunk, freed the left gastric artery, splenic artery, and common hepatic artery until the gastroduodenal artery was freed, and completed the arterial trimming.
6. Biliary tract trimming: Cut the common bile duct at the upper edge of the pancreatic head, and flushed the bile duct 4–5 times with 50 ml of normal saline.

### Outcomes

The following outcomes were examined and assessed simultaneously in the liver bench surgery:

1. Whether the effective tissue traction exposure is achieved

The exposure quality can be assessed in terms of “good”, “medium”*,* and “poor*”* according to the assessment from surgeons (Good: Clear exposure, no interruption and no other injury caused by exposure; Medium: fairly good exposure, interruption (caused by exposure problem) less than three times, and no other injury caused by exposure; Poor: Poor exposure, operation interruption (caused by exposure problem) more than three times, and additional damage or injury caused by exposure problem).

(2) The number of assistants needed in the bench operation;
(3) The number of magnetic anchoring traction devices needed in the operation;
(4) The number of tissues injured or damaged in the operation;
(5) The amount of time taken to complete each procedure of surgery

### Statistical methods

The normality test of continuous variables was carried out by Kolmogorov–Smirnov Test. Normally distributed variables (*P* > 0.05) were described in terms of mean ± standard deviation, and T-test was used for hypothesis testing. Non-normally distributed variables (*P* < 0.05) used median (interquartile range) to describe, and were compared by Mann–Whitney rank sum test. Categorical variables were described in terms of counts or percentages, and compared by chi-square test or Fisher's exact test. The inspection level was α = 0.05, which mean that *P* < 0.05 indicated that the difference was statistically significant. All statistical analysis was processed by IBM SPSS (version 23.0).

## Results

### The baseline of MAT group and manual group

The baseline information of donor livers in the MAT group and the manual group were presented in Table 1.

**Table 1:** Baseline data of donor liver between two groups

Variants	MAT group (n = 10)	Manual group (n = 10)	*P* value	Testing methods
*Demographic characteristics*				
Age	52 ± 15	42 ± 14	0.184	t-test
Gender (male/female)	6/4	8/MAT2	0.628	Fisher’s
Height (m)	1.66 ± 0.06	1.68 ± 0.06	0.448	t-test
Weight (kg)	57 ± 7	60 ± 6	0.349	t-test
BMI (kg/m^2^)	20.6 ± 1.4	21.2 ± 1.7	0.438	t-test
*Donor liver status*				
Volume (small, medium, large)	0/9/1	1/8/2	0.739	MWW
Perfusion condition (good, medium, poor)	8/2/0	7/3/0	0.481	MWW
Fatty liver (yes/no)	2/8	1/9	> 0.999	Fisher’s
Cirrhosis (yes/no)	0/10	0/10	> 0.999	Fisher’s
Surgeon (surgeon A/surgeon B)	5/5	6/4	> 0.999	Fisher’s

Ten donor livers were processed in each group. The aspects about the age, gender, height, weight, body mass index (BMI), and donor liver state, volume, perfusion, presence or absence of fatty liver or cirrhosis were described and compared between two groups. To avoid the bias caused by different operating surgeons, surgeons between two groups were compared as well in age and entire period of operation time. No statistical difference was found regarding the baseline data between two groups.

### Comparison of surgical procedures between MAT group and manual group

**Fig. 1:**
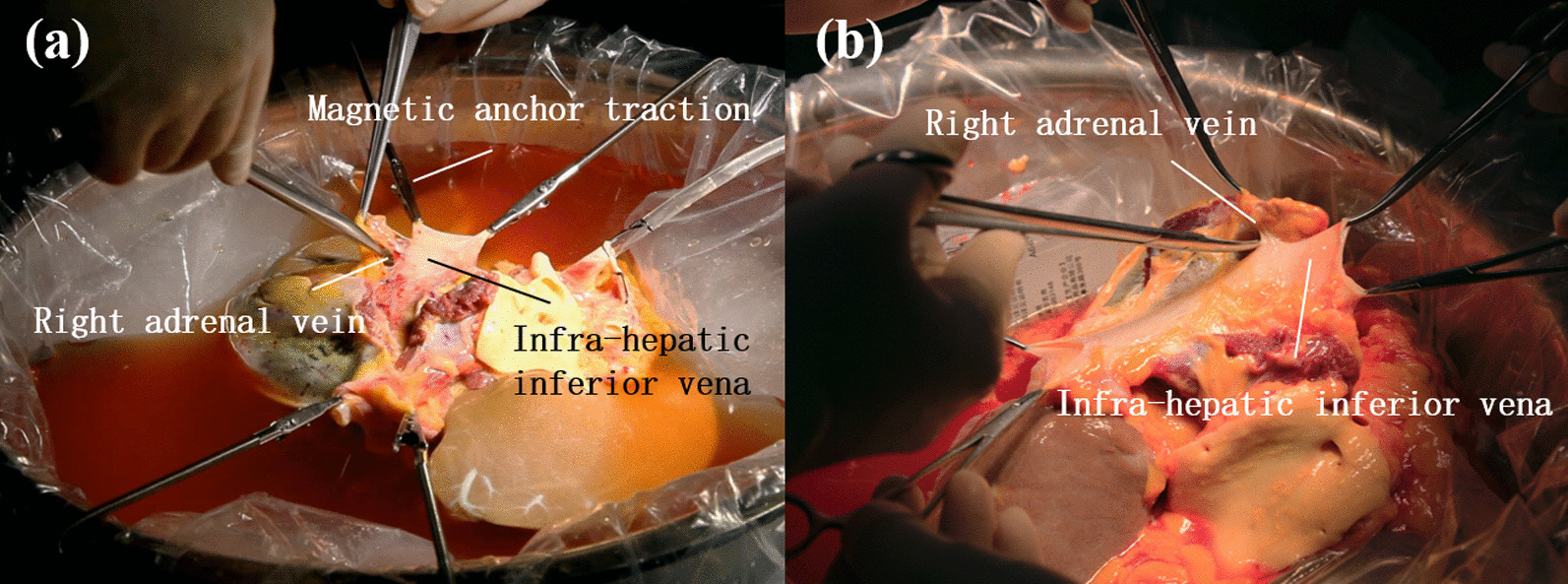
Infra-hepatic inferior vena cava trimming. **a** MAT group **b** Manual group

1. Infra-hepatic inferior vena cava trimming:

The trimming results of infra-hepatic inferior vena cava between two groups were displayed in Table 2 below, along with two trimming surgery photos from each group as presented in Fig. 1. In the MAT group, 4.0 (2.0, 4.0) MAT devices were used in the exposure, reducing the number of manual assistants to 1.0 (1.0, 1.0), less than 1.5 (1.0, 2.0) hands needed in the manual group, and the difference was statistically significant (*P* = 0.023). The exposure quality was assessed 100% “good” for the MAT group, while only six cases were rated “good” for the manual group (60%), even though the difference was not statistically significant. No vascular wall damage occurred in both groups. As for the surgery time, the MAT group took 10 min (9, 16) to complete the procedure, shorter than 22 min (10, 25) spent by the manual group, though the difference was not statistically significant.

(2) Supra-hepatic inferior vena cava trimming

**Table 2:** Results of infra-hepatic inferior vena cava trimming between MAT group and manual group

Variants	MAT group (n = 10)	Manual group (n = 10)	*P* value	Test methods
Number of assistants	1.0 (1.0, 1.0)	1.5 (1.0, 2.0)	0.023*	MWW
Number of magnetic anchoring traction devices used	4.0 (2.0, 4.0)	0		
Exposure quality (good/medium/bad)	10/0/0	6/4/0	0.143	MWW
Vascular wall damage (yes/no)	0/10	0/10		
Events of vascular wall damage occurred	0	0	> 0.999	MWW
Procedure time (min)	10 (9, 16)	22 (10, 25)	0.052	MWW

*P < 0.05

**Fig. 2:**
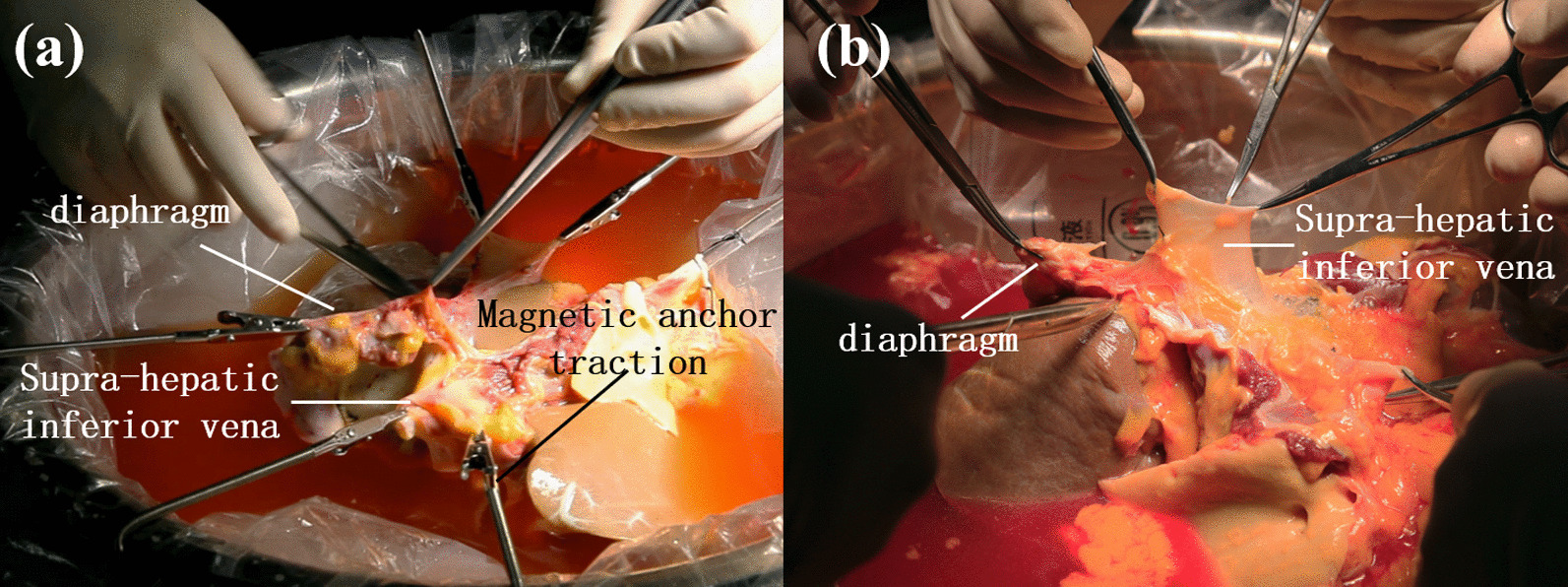
Supra-hepatic inferior vena cava trimming. **a** MAT group **b** Manual group

The results of supra-hepatic inferior vena cava trimming performed by the MAT group and the manual group were presented in Table 3 below, along with photos from two groups as displayed in Fig. 2. The MAT group used 4.0 (2.0, 4.0) MAT devices instead of manual assistance to stretch the inferior vena cava in the exposure of vascular branches, and the number of assistants needed was only 1.0 (0, 1.0), which was significantly less than 1.5 (1.0, 2.0) assistants needed in the manual group (*P* = 0.011). In addition, the exposure quality of ten cases in the MAT group was ranked “good” straight, while only six cases (60%) were rated “good” in the manual group. In the trimming operation, no vascular wall damage occurred in the MAT group, while there were three occurrences of vascular damage in the manual group, and one in each surgery. In the MAT group, it took only ten minutes (9, 13) to complete the procedure, shorter than 28 min (10, 30) taken by the manual group, and the difference was statistically significant (*P* = 0.009).

(3) Portal vein trimming

**Table 3:** Results of supra-hepatic inferior vena cava trimming between MAT group and manual group

Variants	MAT group (n = 10)	Manual group (n = 10)	*P* value	Test methods
Number of assistants	1.0 (0, 1.0)	1.5 (1.0, 2.0)	0.011*	MWW
Number of magnetic anchoring traction devices used	4.0 (2.0, 4.0)	0		
Exposure quality (Good/Medium/Bad)	10/0/0	6/1/3	0.143	MWW
Vascular wall damage (yes/no)	0/10	3/7	0.211	Fisher’s
Events of vascular wall damage occurred	0	0 (0, 1.0)	0.280	MWW
Procedure time (min)	10 (9, 13)	28 (10, 30)	0.009*	MWW

*P < 0.05

**Fig. 3:**
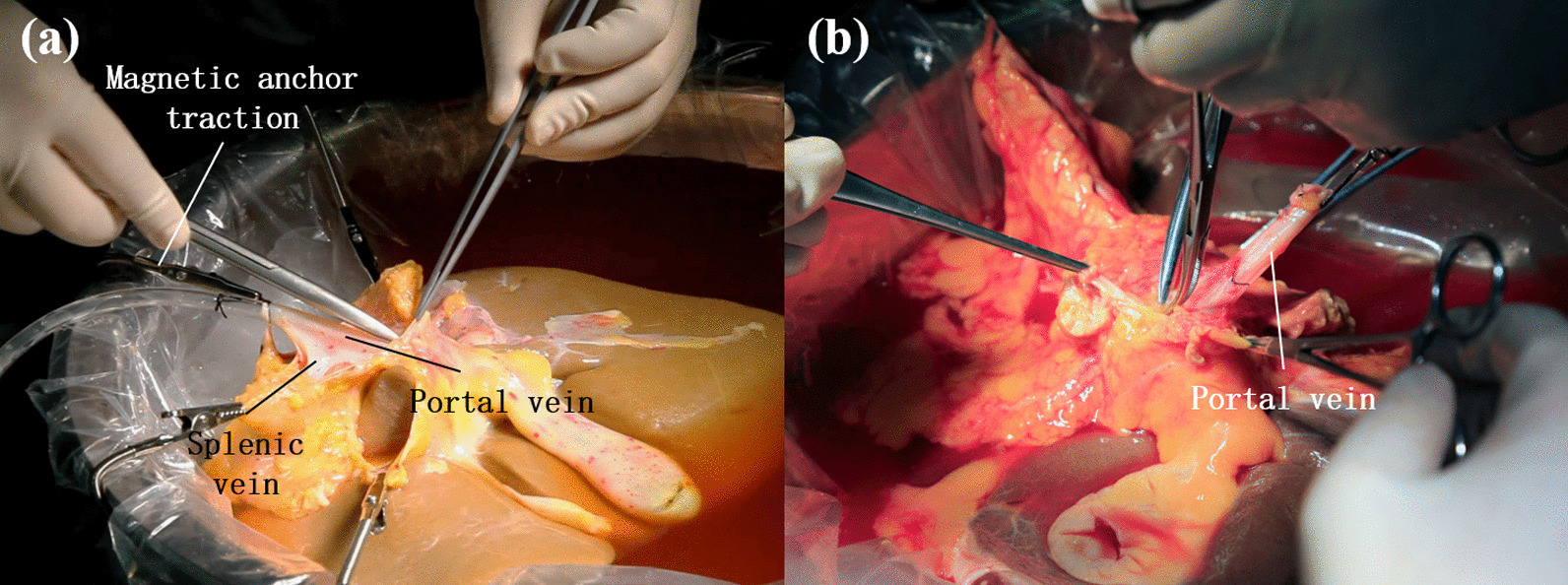
Portal vein trimming. **a** MAT group **b** manual group

The results of portal vein trimming in the MAT group and the manual group were displayed in Table 4, along with photos from two groups as presented in Fig. 3. In the surgery, 2.0 (2.0, 2.5) MAT devices instead of manual assistance were employed to expose the portal vein and its branches, so the number of hands needed in the MAT group was 1.0 (1.0, 1.0), significantly less than 1.5 (1.0, 2.0) assistants needed in the manual group (*P* = 0.035). When it comes to the exposure quality, nine cases of portal vein trimming in the MAT group were rated “good”, and the left one failed to find a portal vein branch due to limited exposure, resulting in the venous wall damage. There were eight cases in the manual group rated “good” exposure, and no vascular wall damage occurred. As for the surgery time, the MAT group took 15 min (10, 15) to complete the operation compared with 15 min (10, 20) taken by the manual group, with no significant difference between two groups.

(4) Hepatic artery trimming

**Table 4:** Results of portal vein trimming between MAT group and manual group

Variants	MAT group (n = 10)	Manual group (n = 10)	*P* value	Test methods
Number of assistants	1.0 (1.0, 1.0))	1.5 (1.0, 2.0)	0.035*	MWW
Number of magnetic anchoring traction devices used	2.0 (2.0, 2.5)	0		
Exposure quality (good/medium/bad)	9/1/0	8/2/0	0.739	MWW
Vascular wall damage (yes/no)	1/9	0/10	> 0.999	Fisher’s
Events of vascular wall damage occurred	0 (0, 0)	0	0.739	MWW
Procedure time (min)	15 (10, 15)	15 (10, 20)	0.436	MWW

*P < 0.05

**Fig. 4:**
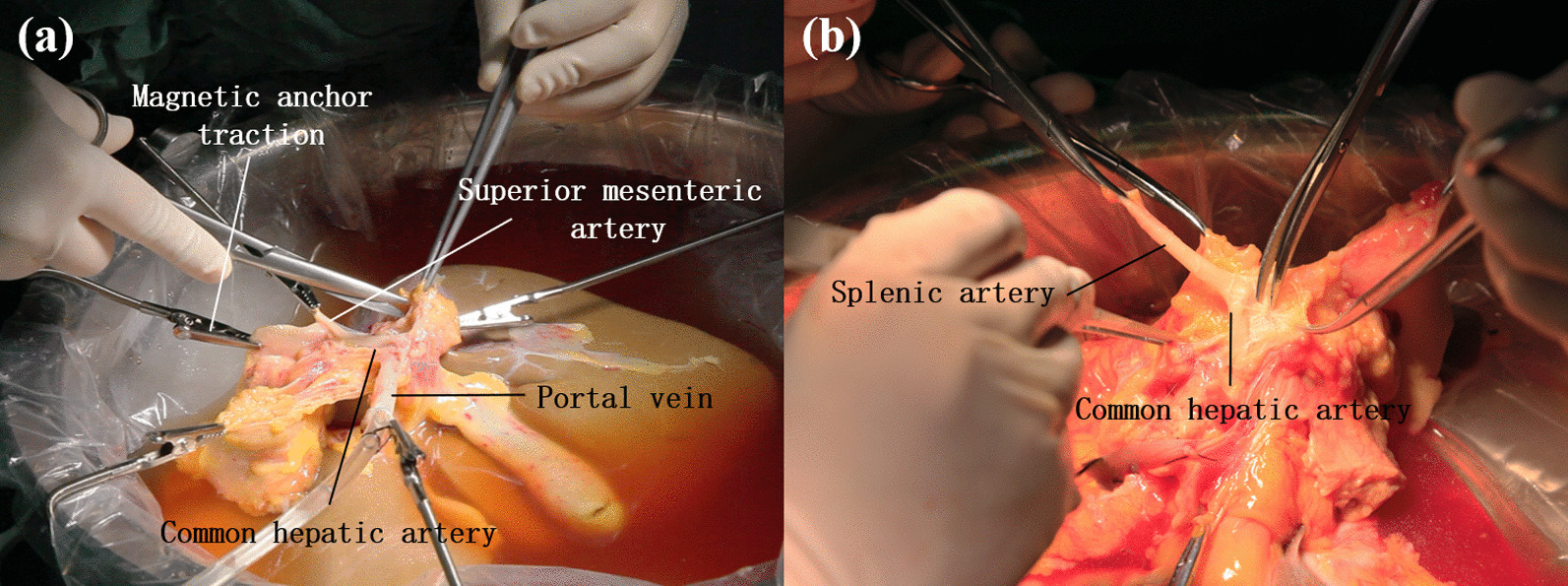
Hepatic artery trimming. **a** MAT group **b** manual group

The results of hepatic artery trimming in the MAT group and the manual group were demonstrated in Table 5 below, along with trimming photos from two groups as presented in Fig. 4. In the MAT group, 2.0 (2.0, 2.5) MAT devices were employed to completely replace the manual assistant to achieve the exposure of hepatic artery, compared with 1.5 (1.0, 2.0) hands needed in the manual group, and the difference was statistically significant (*P* < 0.001). The exposure quality of ten cases in the MAT group reached good level according to the assessment of surgeons, while barely six cases (60%) of exposure were rated “good” in the manual group. No vascular wall damage occurred in both groups in the trimming. Finally, a significant difference in surgery time was there between two groups: The MAT group took only 15 min (10, 20) to complete the procedure, significantly shorter than 20 min (20, 30) taken by the manual group.

(5) Biliary tract trimming

**Table 5:** Results of hepatic artery trimming between MAT group and manual group

Variants	MAT group (n = 10)	Manual group (n = 10)	*P* value	Test methods
Number of assistants	0 (0, 0)	1.5 (1.0, 2.0)	< 0.001*	MWW
Number of magnetic anchoring traction devices used	2.0 (2.0, 2.5)	0		
Exposure quality (good/medium/bad)	10/0/0	6/4/0	0.143	MWW
Vascular wall damage (yes/no)	0/10	0/10		
Events of vascular wall damage occurred	0	0	> 0.999	MWW
Procedure time (min)	15 (10, 20)	20 (20, 30)	0.023*	MWW

*P < 0.05

The results of biliary tract trimming by the MAT group and the manual group were displayed in Table 6 below. Because the trimming is relatively simple in its operation, the surgeon can complete it on his own. No MAT device was employed by two groups, so there was no difference in the use of manual assistance, as well as in the exposure quality and biliary tract injury. As for the surgery time, there was little difference between two groups considering the fact that the MAT group took six minutes (5, 10) to complete the trimming, in contrast to ten minutes (7, 10) spent by the manual group.

(6) Comparison of overall surgery results between two groups

**Table 6:** Results of biliary tract trimming between two groups

Variants	MAT group (n = 10)	Manual group (n = 10)	*P* value	Test methods
Number of assistants	0	0	> 0.999	MWW
Number of magnetic anchoring traction devices used	0	0		
Exposure quality (good/medium/bad)	10/0/0	10/0/0	> 0.999	MWW
Vascular wall damage (yes/no)	0/10	0/10		
Events of vascular wall damage occurred	0	0	> 0.999	MWW
Procedure time (min)	6 (5, 10)	10 (7, 10)	0.218	MWW

*indicates *P* < 0.05

The overall surgery results between two groups were presented in Table 7 below. In the MAT group, 4 (2, 4) MAT devices were used for each liver bench surgery, reducing the number of manual assistants to 1.0 (1.0, 1.0), compared with 1.5 (1.0, 2.0) assistants needed in the manual group, and the difference was statistically significant (*P* = 0.035). Taking the worst exposure effect in all steps as the overall exposure effect, the overall exposure effect of MAT group was 90% as a good level, 10% as a medium level. In manual group, 40% as a good level, 30% as a medium level, and 30% as a poor level. There was a statistically significant difference between the two groups (*P* = 0.043). In the trimming operation, there was only one occurrence of portal vein injury in the MAT group compared with three occurrences of injury in the supra-hepatic inferior vena cava in the manual group. However, the statistical testing displayed little difference in the risk of additional tissue injury or damage between two groups. In addition, no statistical difference was found between two groups in the surgery time to complete the procedure: The MAT group took 55 min (51, 71) to complete the liver bench, against 85 min (54, 85) spent by the manual group.

(7) Completion of bench surgery

**Table 7:** Results of overall surgery between MAT group and manual group

Variants	MAT group (n = 10)	Manual group (n = 10)	*P* value	Test methods
Number of assistants	1.0 (1.0, 1.0)	1.5 (1.0, 2.0)	0.035*	MWW
Number of magnetic anchoring traction devices used	4 (2, 4)	0		
Exposure quality (Good/Medium/Bad)	9/1/0	4/3/3	0.043*	MWW
Vascular wall damage (Yes/No)	1/9	3/7	0.582	Fisher’s
Events of vascular wall damage occurred	0 (0, 0)	0 (0, 1)	0.481	MWW
Procedure time (min)	55 (51, 71)	85 (54, 85)	0.247	MWW

*P < 0.05

**Fig. 5:**
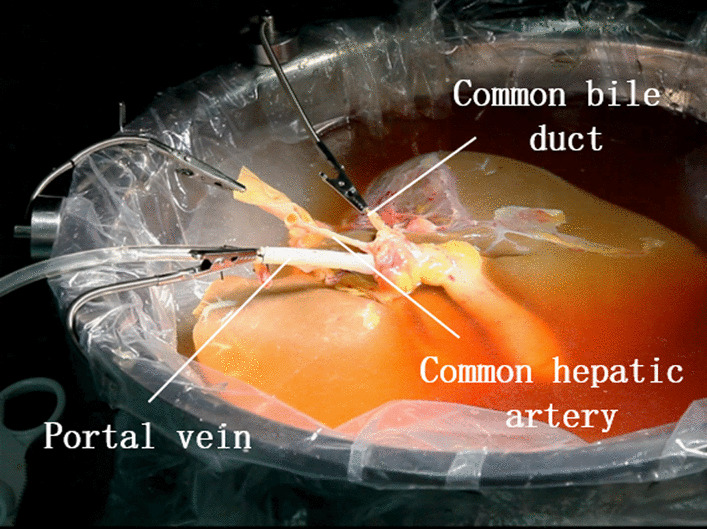
Completion of liver bench surgery

As displayed in Fig. 5 below, the liver bench surgery was completed after the trimming of the infra- and supra-hepatic inferior vena cava, portal vein, hepatic artery and biliary tract.

### Assessment by liver transplant specialists

The application of the MAT system in the clinical treatment was assessed by five liver transplant specialists, and the details were provided in Table 8 below. Besides the advantages of being user friendly and accessible in operation, all five experts agreed that MAT is capable of replacing some manual operations in the liver trimming to enhance traction exposure. Four experts believed that MAT can provide a clearer and even wider vision in the operation, and function as safely as those of the conventional surgical instruments in the performance. While one expert did not think much of the difference between two groups, worrying that MAT might attract other ferromagnetic surgical instruments as well, and which in turn can be a possible problem for the safety of operation. In general, the clinical potentials of the MAT device were rated 2.6 ± 0.24 (out of 3 points) by all specialists.

**Table 8:** Assessment result

Assessment items	Expert 1	Expert 2	Expert3	Expert 4	Expert 5
Convenience	Yes	Yes	Yes	Yes	Yes
Exposure quality	Clear	Medium	Clear	Clear	Clear
Safety	Safe	Safe	Medium	Safe	Safe
Clinical application potential	3	2	2	3	3

### The learning curve for the use of MAT

**Fig. 6:**
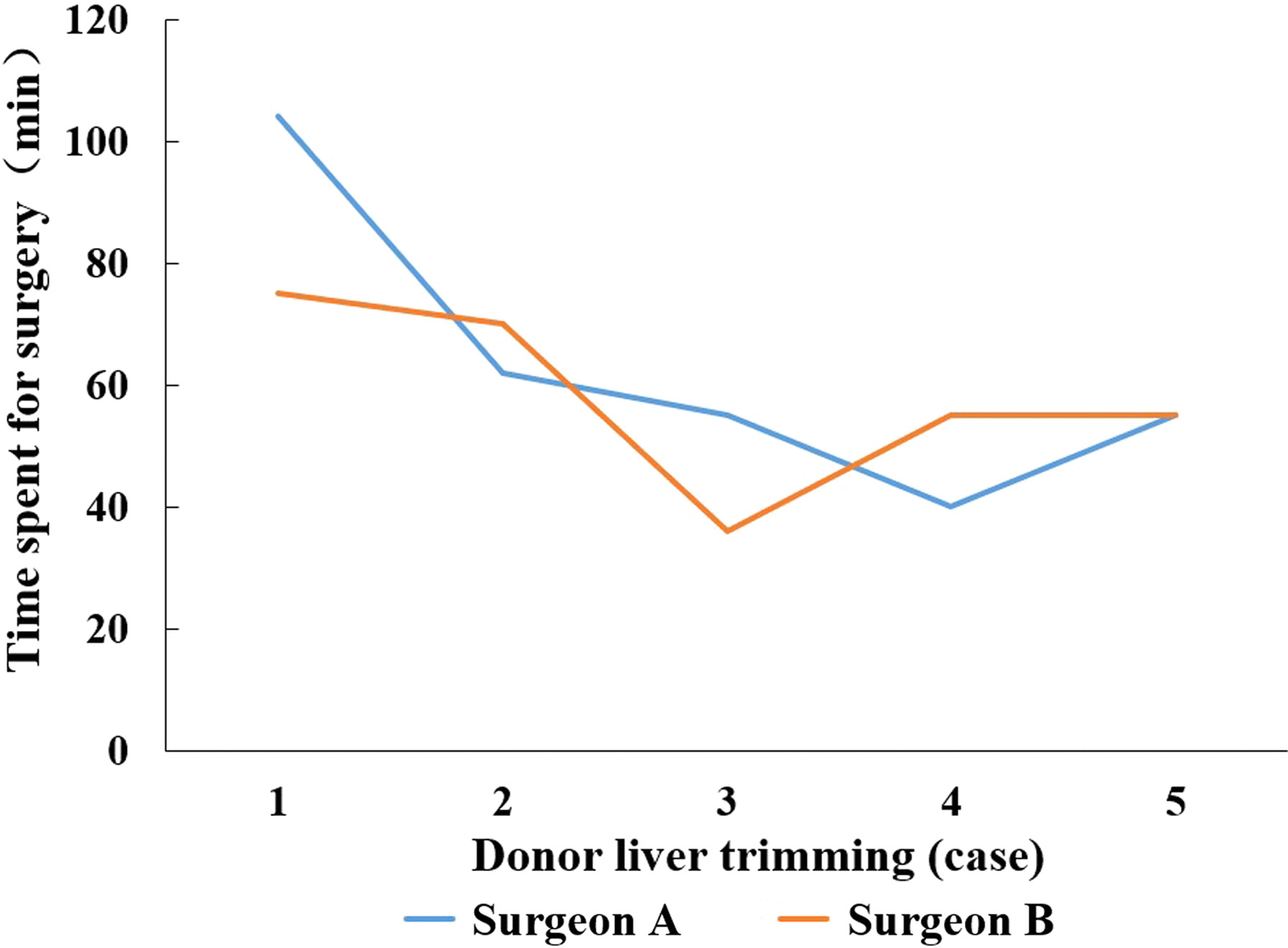
Learning curve for MAT system

In the MAT group, surgeon A and surgeon B completed five liver bench surgery respectively. The surgery time spent by surgeon A in his first try of MAT assisted device was 104 min, and the time shortened significantly from his second surgery on, with an average time around 53 ± 9 min. The time taken by surgeon B in his first and second MAT assisted surgery was 75 min and 70 min, respectively, and the average time for his 3rd to 5th surgery was 49 ± 11 min. After surgeon A and surgeon B rapidly grasped the MAT skills, their surgery time for liver bench shortened accordingly, showing a downward curve as presented in the Fig. 6 below.

### Cost

The MAT device is made up of some reusable and disposable components, which is readily available and inexpensive. The reusable parts, including the magnetic base and dedicated surgical instruments, cost only 30,00 RMB (approximately US$ 470). The disposable parts including four magnetic anchor grippers cost nearly 300 RMB (approximately US$ 47).

## Discussion

As more mechanical devices are applied to the clinic, we developed the MAT system hoping to be a turning-points of liver transplantation. Compared with the assistant, the use of the mechanical system not only increases the field of view, the operation is more precise, and the hand tremor is reduced compared with the human. The overall clinical application indicates that the MAT system can be employed not only to replace manual assistance in the operation, but more importantly enhance the exposure quality without causing additional injury or damage in the surgery.

In the animal experiments, MAT functions perfectly in the stretch exposure of the blood vessels, biliary tract, diaphragm and other tissues, substantially reducing the risk of additional injury. On the basis of these trials, we carry out a succession of MAT assisted liver bench surgery to further verify and address the feasibility, safety and effectiveness of its clinical application in the treatment of liver disease.

With its stable, simple, and reliable features, MAT can be employed readily to replace manual assistance in the liver bench trimming operation. Compared with the conventional approach and equipment, the lasting fixation achieved by the MAT structure can help avoid other injury or damage that might have otherwise been caused by manual operation errors [12–14]. In addition, it is accessible and user friendly, making it a better option for the surgical training. These advantages are welcomed by the specialists and viewed as a great potential for an even broader clinical application.

One of the striking advantages of MAT lies in its stability. As a simple and reliable mechanical system, MAT can complete the exposure perfectly under the total control or supervise of the surgeon, and promote the safety of operation simultaneously. Specifically, the use of MAT makes it possible for the surgeons to dissect arteriovenous and other tissues stably and carefully to achieve a quick and precise repair even in the case of some other injury. This is the way especially in the exposure of inferior vena cava, where good exposures (the ratio of well-exposed cases / total cases) boost from 60 to 100%. In the liver bench surgery, no vascular damage occurred compared with three occurrences of damage in the manual group. When it comes to surgery time, the average operating time (from first touch of the inferior vena cava to the start of next vessel trimming) significantly shortened from 28 min to barely ten minutes (before trimming the blood vessel, the surgery time for adjusting and fixing MAT was recorded separately, and not included in the operating time, while the time for adjusting during the operation was included.)

The MAT system can eliminate the errors possibly caused by the manual operations, thus reducing the traction damage to blood vessels. In the clinical treatment, the number of vascular breaks in two groups is different, even though it is not statistically significant. We can attribute this difference to the stability and reliability of the mechanical structure of MAT. As it is known in the operation, the physical or mental state of assistants, manual errors, or even slight hand tremors may affect the quality of operation, and cause some damage in the trimming blood vessels. In this sense, we consider the lack of statistical difference may to a large extent attribute to the inaccurate specification and classification of injury or damage. In addition, the lack of enough samples may also be a possible explanation for the result. In the trimming of portal vein and biliary tract, the difference between two groups in the exposure quality, vascular damage and operation time was not obvious, which may be related to less soft tissue wrapping and clearer surrounding anatomical structure. In the process of biliary tract trimming, since the operation is simple, only the common bile duct needs to be cut at the upper edge of the pancreatic head after the biliary tract flushing, the surgeon can complete this operation independently. The assistant surgeon, sometimes considered unnecessary, should be well aware of the chief surgeon’s operations and help to maintain a steady and stable exposure. Our clinical practices indicate that the MAT device can achieve a better exposure without causing additional damage to the donor liver compared with the manual operation.

With its striking advantages of time saving, easy learning, and being user friendly, the MAT system can develop into a new front in the liver bench trimming, and generate a profound impact on the progress of liver transplantation. Compared with the longer learning time for the conventional trimming approach, the MAT system renders it possible for the surgeons to quickly grasp the key operating skills, saving lots of time and labor in the surgery as a result of it [15–17].

Using the MAT system, we performed 20 liver bench surgery and compared the amount of time spent for surgery between the MAT assisted group and the manual group. The results indicate a significant difference (*P* = 0.019) in the time taken for the donor liver trimming between two groups considering the fact that the total surgery time spent by MAT assisted group is 55 min (40, 55), in contrast to 85 min (54, 85) taken by its counterpart group. When it comes to the training, the result indicates that two surgeons can quickly grasp the MAT skills after only two surgical practices, significantly shortening the amount of time that would have otherwise been spent in the learning of conventional donor liver trimming by more than 30%.

The MAT system can also be employed by surgery learners or trainees themselves in the animal liver trimming practice, thus efficiently promoting the learning and largely reducing the training cost.

Since the MAT assisted liver trimming usually requires only one assistant, a surgeon can rely entirely on his own to perform whole procedures in shortage of hands, thus reducing the labor cost. In the preliminary animal experiments, altogether 25 MAT assisted donor liver trimmings were performed by a single surgeon. In the clinical application, there was a case of donor liver retrieval completed by a single surgeon and four MAT devices due to the shortage of assistant at the time. The use of MAT alleviates the urgent problem of the shortage of professional and technical personnel to a certain extent. Our original intention is by no means completely replace, nor to eliminate, assistants or guidance from experienced surgeons. On the contrary, the application of the MAT system allows junior one to grow up in the shortest time. In practice, the MAT system is capable of replacing manual labor in the procedures of infra-hepatic vena cava, supra-hepatic vena cava, portal vein and hepatic artery (which often need 1–2 supporting hands in the conventional bench surgery) and achieving better exposure and trimming effect.

Five liver transplant specialists performed the MAT assisted liver bench surgery. Most of them speak highly of the system, believing that it is a robust and reliable assisting alternative, and can provide an even clearer vision in the surgical performance. Still one expert is concerned about the safety problem, questioning that it may attract other ferromagnetic surgical instruments in the operation, and suggesting the use of some anti-magnetic materials instead. Another worry is about the possible injury on tissue due to the forceps gripping. We are making an effort to solve these possible problems through a wider co-operation with other specialists in the hope to reduce the risk to the minimum. Others have suggested improvements to our methodology. At the time of the experiment, the only equipment we had was insufficient to support full randomization. A period of time which the MAT system was required for surgical grade sterilization and return. We are still in the stage of testing efficacy and safety of MAT system. Baseline data that may affect operative time and the occurrence of co-injury were consistent, especially organ size and fatty liver. (Table 1) This alleviates the methodological anxiety to some extent. The former greatly affects the prolongation of the operation time, and the latter may lead to differences in the probability of injury due to the fragility of the liver.

In summary, as a stable and reliable assistance in the surgery, the novel MAT system can be employed effectively in the liver bench trimming surgery as well as in the training of liver transplant specialists. In the clinical application it has displayed many advantages such as labor and time saving, risk minimizing, and user friendly, which in turn can help to enhance the quality of surgical treatment. These benefits are increasingly valued by the professionals and specialists, and can be extended into an even wider areas of medical care. In the future research, we plan to conduct further research to improve the use of the MAT system in the hope of providing more evidence and information about its application in the clinical treatment and liver transplantation.

## Data Availability

The datasets generated and/or analyzed in the experiment are available from the corresponding author on reasonable request.
